# Full Mitochondrial Genome Sequence of the Sugar Beet Wireworm *Limonius californicus* (Coleoptera: Elateridae), a Common Agricultural Pest

**DOI:** 10.1128/genomeA.01628-15

**Published:** 2016-01-21

**Authors:** Alida T. Gerritsen, Daniel D. New, Barrie D. Robison, Arash Rashed, Paul Hohenlohe, Larry Forney, Mahnaz Rashidi, Cathy M. Wilson, Matthew L. Settles

**Affiliations:** a Institute for Bioinformatics and Evolutionary Studies (IBEST), University of Idaho, Moscow, Idaho, USA; b Department of Biological Sciences, University of Idaho, Moscow, Idaho, USA; c Department of Plant, Soil and Entomological Sciences, University of Idaho Aberdeen R&E Center, Aberdeen, Idaho, USA; d Idaho Wheat Commission, Boise, Idaho, USA

## Abstract

We report here the full mitochondrial genome sequence of *Limonius californicus*, a species of click beetle that is an agricultural pest in its larval form. The circular genome is 16.5 kb and contains 13 protein-coding genes, 2 rRNA genes, and 22 tRNA genes.

## GENOME ANNOUNCEMENT

Wireworms are the larval stage of click beetles (Coleoptera: Elateridae), of which several species are serious pests due to their damage to the seedlings, roots, and stem tissues of economically important crops (1, 2). The sugar beet wireworm *Limonius californicus* (Mannerheim) is a significant threat to cereal and potato production in the Pacific Northwest region of the United States. Control methods for larvae were developed in the 1950s and involved the wide application of environmentally persistent chemicals, such as dichlorodiphenyltrichloroethane (DDT) and γ-hexachlorocyclohexane (Lindane) (3), both of which were later banned for use and are not used on crops today due to health and environmental risks. Although current broad-spectrum insecticides, such as neonicotinoids, induce morbidity, wherein the larvae become sick and stop feeding for a period, they subsequently survive to feed and eventually reproduce (3, 4). Constructing a genetic reference for this pest is not only the first step in creating more targeted control methods but also essential in understanding how populations evolve resistance in response to pesticides.

A single specimen of *L. californicus* was collected on 10 December 2014 from a farmer’s field in Aberdeen, ID, at a 6-inch depth into the soil. The specimen species was confirmed on site, and DNA extraction was conducted using cetyltrimethylammonium bromide (CTAB) methodology. The DNA was sheared to 800-bp average fragment length, and library preparation was performed on the Apollo 324 platform. Reads were sequenced on an Illumina MiSeq with paired-end 300-bp lengths at the University of Idaho’s IBEST Genomics Resources Core; sequencing of the library resulted in 160× coverage of the mitochondrial genome.

Following sequencing, reads were processed using a custom bioinformatics pipeline to remove duplicate reads, trim off low-quality bases and sequence adapters from the read ends, and overlap pairs using FLASH (GitHub) (5). The mitochondrial genome was then assembled using the ARC software package (GitHub), which uses a mapping reference to seed for iterative assemblies. The *Elateridae* species (Coleoptera: Elateridae; accession number KF961574) mitochondrial genome sequence was used to initially seed the ARC assembly.

Following assembly, NCBI BLAST was used to identify a large set of related mitochondrial sequences from coleopteran species and assemble a tree using FastTree (6). Gene annotations were predicted from the MITOS Web server (7); annotations and open reading frames (ORFs) were later refined and confirmed by constructing a custom BLAST database in Geneious 8.0 (8) from the reference species list. A total of 51 species were compared to the *L. californicus* assembly, with *Chaetosoma scaritides* (Coleoptera: Chaetosomidae; accession no. NC_011324) identified as the closest sequence.

The mitochondrial DNA (mtDNA) genome of *L. californicus* is a circular DNA molecule of 16,783 bp, with a G+C content of 29.5%. Predicted annotations from both Geneious and the MITOS server include common respiratory genes (*atp6, atp8, cob, cox1, cox2, cox3, nad1, nad2, nad3, nad4, nad4l, nad5,* and *nad6*), 2 rRNA genes (large and small subunits), and 22 tRNA genes. This full mtDNA genome sequence is an important first step in assessing the genetic variation within this pest species and developing targeted control methods.

### Nucleotide sequence accession number.

The mtDNA genome sequence is deposited in GenBank with accession number KT852377***.***
